# Costunolide Induces Autophagy and Apoptosis by Activating ROS/MAPK Signaling Pathways in Renal Cell Carcinoma

**DOI:** 10.3389/fonc.2020.582273

**Published:** 2020-10-26

**Authors:** Dian Fu, Ding Wu, Wen Cheng, Jianping Gao, Zhengyu Zhang, Jingping Ge, Wenquan Zhou, Zhenyu Xu

**Affiliations:** Department of Urology, Jinling Hospital, Medical School of Nanjing University, Nanjing, China

**Keywords:** costunolide, renal cancer, ROS – reactive oxygen species, apoptosis, autophage

## Abstract

Although costunolide (Cos), a natural sesquiterpene compound isolated from various medicinal plants, exhibits antiproliferative and pro-apoptotic effects in diverse types of cancers, the mechanism associated with the anticancer property of Cos has not been elucidated. The present investigation was carried out to study the anticarcinogenic influence of Cos on kidney cancer cells. Several human renal cancer cell lines were used and biological and molecular studies were conducted. It was found that Cos significantly suppressed renal carcinoma cell growth *via* stimulation of apoptosis and autophagy in a concentration-dependent manner. Further studies revealed that Cos increased Bax/Bcl-2 ratio, decreased mitochondrial transmembrane potential (MMP), and enhanced cytoplasmic levels of cytochrome *c*, and activation of caspase-9, caspase-3, and cleaved PARP, resulting in cell apoptosis. The autophagy induced by Cos resulted from the formation of GFP-LC3 puncta and upregulation of LC3B II and Beclin-1 proteins. Compared with Cos treatment, the autophagy inhibitor 3-MA or ROS scavenger NAC significantly inhibited apoptosis and autophagy. Moreover, NAC and JNK-specific inhibitor SP600125 attenuated the effect of Cos. Taken together, Cos exerted autophagic and apoptotic effects on renal cancer through the ROS/JNK-dependent signal route. These findings suggest that Cos could be a beneficial anticarcinogenic agent.

## Introduction

Renal cell carcinoma (RCC) comprises 3–4% of all human cancers, and it is the most lethal kidney malignant tumor (1). Surgical intervention is the most effective therapeutic strategy for RCC. However, up to 30% of RCC cases are diagnosed at the stage of metastasis (2). The 5-year overall survival of metastatic RCC patients is below 10%. Typically, RCC is insensitive to traditional chemo- and radio-therapeutic treatments (3). Moreover, the use of targeted treatment options as first and second-line treatments have limited effect on the survival rates. Therefore, there is need for exploring low-toxicity novel treatment strategies for RCC.

Costunolide (Cos) is a naturally occurring sesquiterpene compound present in various medicinal plants, including *Magnolia sieboldii*, *L aurus nobilis*, and *Saussurea lappa* (4, 5). It has various effects such as anti-inflammatory and antifungal properties (6, 7). Recently, Cos has been reported to be able to assist chemotherapeutic agents in overcoming multidrug resistance in cancer cells (8). Although some studies have shown that Cos exhibits potent anticarcinogenic activity in human cancer cells through induction of cell cycle arrest and apoptosis (9, 10), its effect on human renal cancer cells and the possible associated mechanisms have not been unraveled.

Cell death can be classified according to the classical morphological criteria as apoptotic or autophagic. Apoptotic cell death is a tightly regulated event, which is important for sustaining tissue constancy *via* removal of genetically compromised cells. The typical features of apoptosis are membranous blebs and nuclear fragments (11). It has been established that apoptosis may occur through either extrinsic or intrinsic route (12). Both pathways may lead to the activation of a related group of caspases involved in the initiation (caspases-8 and -9) and execution (caspases-3) phases of apoptosis (13). Autophagy is an evolutionarily preserved process by which cells degrade macromolecules, unwanted organelles and certain types of bacteria *via* double-membrane structures termed autophagosomes (14). Autophagy performs a complex function in cancer development and treatment (15). It can function as a cytoprotective mechanism that protects cancer cells from apoptotic cell death induced by various anticancer drugs (16). On the other hand, excessive autophagy can cause cell death and arrest tumor progression. Therefore, extensive attention has been paid to redefining the precise function of autophagic processes in malignancy therapy, so as to enhance the designing, selection, and utilization of autophagy-regulating agents (autophagy inducers or inhibitors) (17). In addition, increasing evidence have shown that apoptosis and autophagy may be cooperative or antagonistic to determine cell fate depending on cell types, strength, and duration of the stress-inducing signals, and influence of other signaling routes (18).

In this study, it was found that Cos exerted reactive oxygen species (ROS)-induced autophagic and apoptotic effects on renal cancer cells through ROS induction, resulting in stimulation of JNK signal pathway. Thus, Cos could be a promising inducer of autophagy and apoptosis, which can be used for targeting human cancers.

## Materials and Methods

### Materials and Chemicals

Cos, 3-methyladenine, and inhibitors of JNK, MAPK, and ERK1/2 were purchased from Selleck. Cos was dissolved in dimethyl sulfoxide (DMSO) and preserved at –20°C. RPMI-1640, DMEM, and FBS were products of Thermo Fisher, while N-acetyl-L-cysteine was obtained from Sigma (St. Louis, MO, United States). Immunoglobulins against caspases-3, -9, and -8; and Bax, PARP, Bcl-2, Cyt c, CoxIV, JNK, p-JNK, p38, p-p38, ERK, phospho-ERK, LC3B, Beclin-1, and β-actin were products of Cell Signaling Technology (Shanghai, China). Reagents for mitochondrial transmembrane potential (MMP) and apoptosis were obtained from Beyotime Inst. Biotech (Beijing, China). Polyvinylidene difluoride membrane was product of Millipore Corp, United States.

### Cell Maintenance and Cultural Conditions

Four human RCC cells (786-O, A-498, ANCH, and 769-P) were supplied by American Type Culture Collection (Manassas, Virginia, United States). The cell lines were cultured in medium (786-O and 769-P in RPMI-1640; A-498 and ANCH in DMEM) with fetal calf serum and antibiotics. The cell culture was done in a 37°C and 5% CO_2_ humidified atmosphere. The cells were grown to confluence before drug treatment. Cos was solubilized in DMSO.

### Cell Viability Assay

The CCK8 assay was used. The cells in suspension were exposed to graded doses of Cos (5, 10, 20, and 40 μM) for 24 h, followed by incubation with 10 μL CCK8 solution for 180 s at 37°C and measurement of absorbance at 455 nm.

For cell counting, cell suspension was incubated for 24 h with the same doses of Cos as in CCK8 assay. Thereafter, the population of dead cells was determined with trypan blue dye exclusion procedure.

### Nuclear Morphologies of Apoptotic Cells

Cell suspension treated with graded doses of Cos were subjected to fixation in paraformaldehyde and stained with DAPI away from light. Nuclear fluorescence intensities were obtained using Nikon fluorescence microscopy (Nikon Inc., Japan).

### Flow Cytometry Analysis of Apoptosis

After treatment with Cos, the cells were rinsed in phosphate-buffered saline (PBS) and resuspended in 200-μL binding solution that contained 5 μL Annexin V-FITC and 10 μL propidium iodide for 20 min away from light. All samples were subjected to flow cytometric analysis.

### Caspase Activity Assay

Caspase-3, caspase-8, and caspase-9 were assayed fluorometrically using Beyotime kits (Beijing, China) in line with respective manual protocols.

### Measurement of Mitochondrial Transmembrane Potential

The MMP (Δψm) was measured using JC-1 Assay Kit (Beyotime, Beijing, China) in line with the manufacturer’s protocol. The Cos treatment was followed with JC-1 staining for 30 min away from light at 37°C. Cellular fluorescent photographs obtained with microscope (Nikon Inc., Japan) were analyzed with flow cytometry.

### RNA Isolation and Real-Time Quantitative PCR Assays

The 769-P cells were plated in six-well plates. After 12-h incubation, the cells were exposed to Cos (10, 20, and 40 μM) for 24 h, followed by extraction of total RNA and cDNA generation, and RT-PCR with Bio-Rad iQ5 System, with β-actin as control. The β-actin primers were generated as outlined previously (19). Relative abundances of the target mRNAs were calculated.

### ROS Generation

The generation of intracellular ROS was determined with a cell-permeable probe, DCFH-DA. Following treatment of the cells with Cos, they were subjected to incubation with the fluorescent probe at 37°C for ½ h away from light. Fluorescent photographs of cells were analyzed with flow cytometry.

### GFP-LC3 Puncta Assay

The 769-P cells with stable expression of GFP-LC3 were plated in six-well plates. After treatment with Cos, the cells were PBS-rinsed and paraformaldehyde-fixed at room temperature for 10 min. Following removal of paraformaldehyde, the cells were washed thrice with PBS and then stained with DAPI away from light at room temperature. Fluorescent images were captured with a fluorescence microscope.

### Western Blotting

Extraction of total protein from Cos-treated 769-P cells followed the method outlined earlier (19). Moreover, proteins from mitochondria and cytosol were extracted using appropriate Kits (Pierce, Rockford, IL, United States). Bicinchoninic assay method was used for determination of protein levels. Then, equal amounts of protein were subjected to 12% SDS-PAGE and electro-transferred to PVDF membranes, the membranes were blocked in 5% non-fat dry milk at room temperature for 1 h, and then incubated with primary antibodies for overnight at 4°C. Thereafter, the membranes were subjected to incubation with HRP-linked 2° conjugated secondary antibodies at room temperature for 1 h. The signals were detected using chemiluminescence (ECL).

### Statistical Analysis

All data are expressed as mean ± SD of three independent experiments. Student’s *t*-test and one-way ANOVA were employed for statistical analyses. Values of *p* less than 0.05 were considered statistically significant.

## Results

### Cos Decreased Cell Viability via Induction of Apoptosis in RCC

**FIGURE 1:**
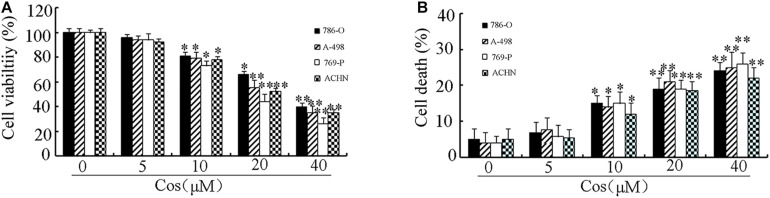
Treatment with Cos decreased cell viability and induced cell death. **(A)** Cell viability, as determined with CCK8 assay. **(B)** Cell death, as measured using trypan blue exclusion assay. All results are presented as mean ± SD (*n* = 3). *p < 0.05; **p < 0.01, vs the control.

**FIGURE 2:**
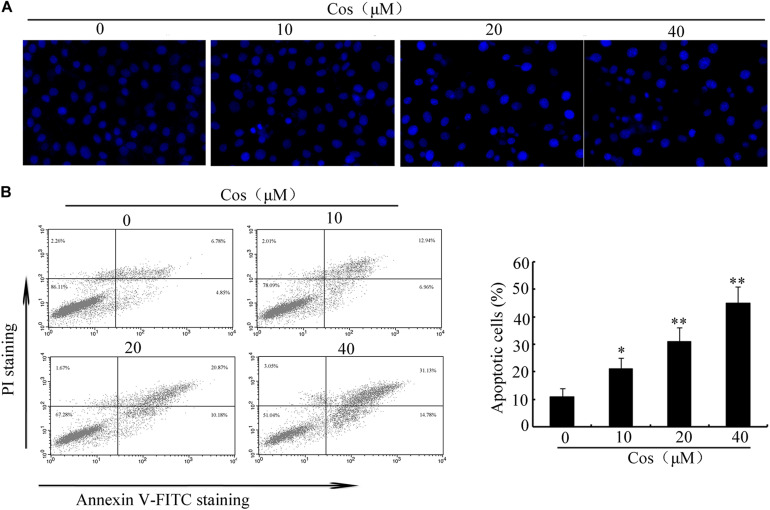
Cos induced apoptosis in 769-P cells. **(A)** Apoptotic nuclear morphology, as assessed using DAPI staining and visualized using fluorescence microscopy. **(B)** Percentage of Cos-induced apoptosis in 769-P cells, as measured using Annexin V-FITC/PI staining and flow cytometry. The histograms indicate the percentage of early apoptosis and late apoptosis. All results are presented as mean ± SD (*n* = 3). *p < 0.05; **p < 0.01, vs the control.

To determine the cytotoxic influence of Cos on RCC cells, the 786-O, A-498, 769-P, and ACHN cells were exposed to different concentrations of Cos (10, 20, and 40 μM) for 24 h, followed by CCK8 and Trypan blue exclusion assays. Figure 1A shows that Cos significantly decreased viability of RCC cells concentration-dependently. Moreover, Cos enhanced apoptosis in a concentration-based fashion (Figure 1B). The occurrence of apoptosis in Cos-treated RCC cells was determined with DAPI staining and flow cytometry. Results revealed that the cells without Cos treatment had rounded nuclei with well distributed chromatin, whereas typical apoptotic features of condensed chromatin and nuclear fragmentation were seen following treatment with Cos (10, 20, and 40 μM) (Figure 2A). Flow cytometric analysis showed that significant and dose-based increases in apoptotic cell number were observed after Cos exposure (Figure 2B).

**FIGURE 3:**
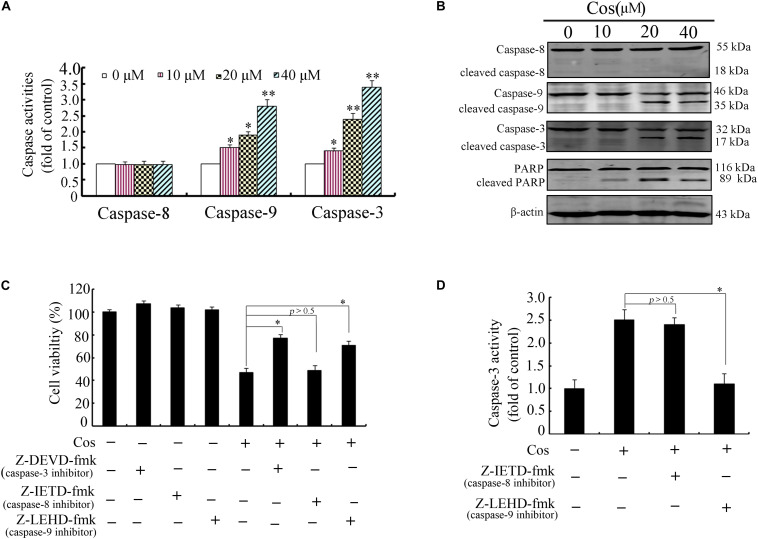
Cos-induced apoptosis was mediated by caspases 9 and 3 in 769-P cells. **(A)** Activities of caspase-3, caspase-8, and caspase-9 as measured using the colorimetric assay kits. **(B)** Caspases-8, -9, and -3, and PARP in 769-P cells treated with various concentrations of Cos for 24 h. **(C)** Effect of caspase inhibitors on Cos-induced cell viability, as measured with CCK8 assay. **(D)** Inhibitory effects of caspase-8 and caspase-9 inhibitors on caspase-3 activity. The activity of caspase-3 was assayed using colorimetric assay kit. All results are presented as mean ± SD (*n* = 3). *p < 0.05; **p < 0.01, vs the control.

Previous studies have demonstrated that apoptosis involves stimulation of cysteine proteases, including both initiators and executors of cell death (13). Thus, further evaluation was done on the effects of Cos on the levels of caspases-8, -9, and -3 using caspase fluorometric assay kits. No significant change was observed in the activity of caspase-8 in the Cos-treated cells, when compared with cells with no Cos treatment. Interestingly, Cos treatment markedly enhanced levels of caspases 9 and 3 (Figure 3A). Consistent with these results, procaspases-9 and -3 levels were lowered with increase in Cos concentration, while the cleaved forms of caspase-9 and caspase-3 increased (Figure 3B). Procaspase-8 was not affected, while PARP was apparently cleaved following Cos treatment for 24 h (Figure 3B). In addition, specific inhibitors of caspase-9 or caspase-3, not caspase-8, significantly attenuated Cos-provoked apoptosis (Figure 3C). Caspase-3 was also significantly inhibited with treatment of caspase-9 inhibitor, but not caspase-8 inhibitor (Figure 3D). The observations provide evidence that Cos enhanced apoptosis *via* stimulation of caspases-9 and -3 only.

### Cos-Activated Mitochondrial Apoptotic Route in RCC Cells

**FIGURE 4:**
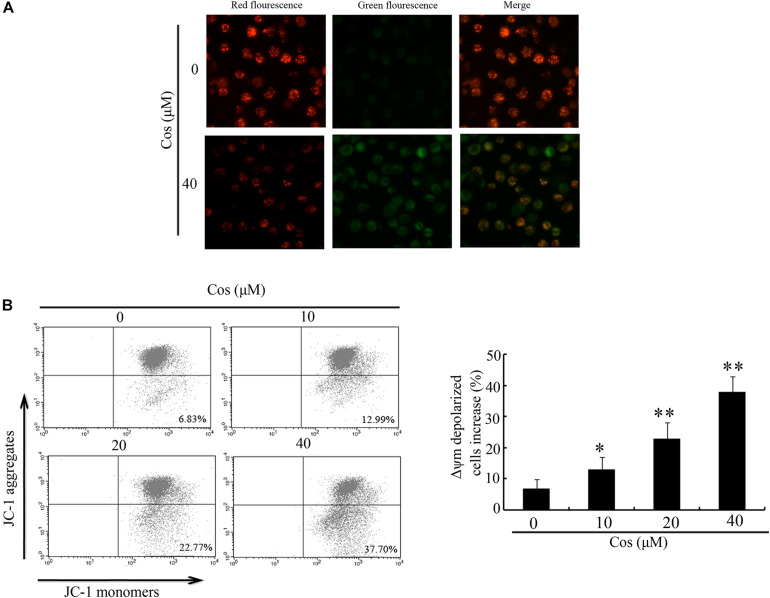
Influence of Cos on mitochondrial membrane potential in 769-P cells. **(A)** 769-P cells exposed to Cos (40 μM) for 24 h and observed under fluorescent microscope after JC-1 staining. Untreated cells served as control. Red fluorescence = normal Δψm; green fluorescence = damaged Δψm. **(B)** Δψm after Cos exposure and JC-1 treatment, as determined flow cytometrically, showing that Δψm-depolarized cells increased by about 26.2 and 61.3% at 12 and 24 h, respectively. All results are presented as mean ± SD (*n* = 3). *p < 0.05; **p < 0.01, vs the control.

To determine whether mitochondrial pathway mediated Cos-induced apoptosis, MMP (Δψm) was determined with JC-1. In normal cells, JC-1 aggregates in normal mitochondria emit red fluorescence. In contrast, JC-1 aggregates in cytosol emit green fluorescence when the mitochondria membrane is depolarized. The results obtained in this study showed a clear change from red to green fluorescence after Cos treatment, indicating that a change in Δψm was triggered by Cos treatment in 769-P cells (Figure 4A). Flow cytometric analysis showed that MMP-depolarized cells were enhanced from 6.83% (normal level) to 12.99, 22.77, or 37.70% after Cos treatment (Figure 4B).

**FIGURE 5:**
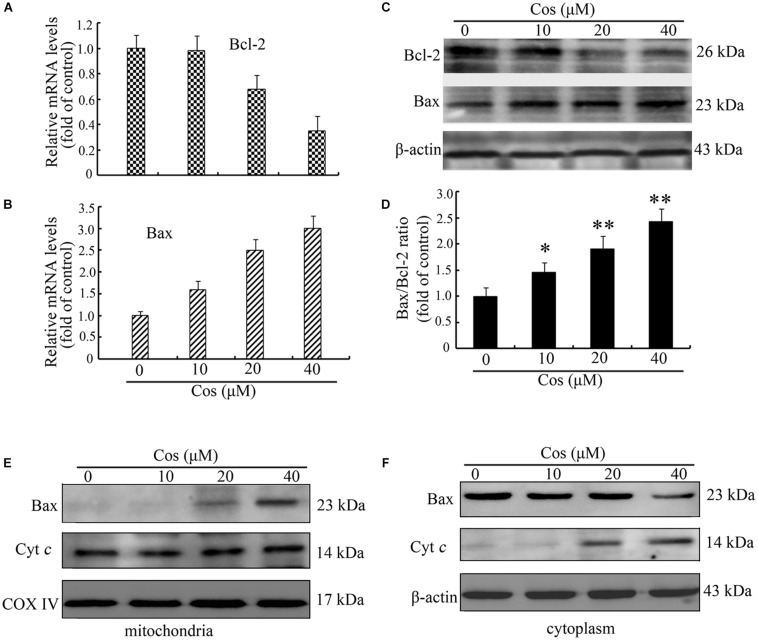
Cos-mediated 769-P cell apoptosis was mediated via mitochondrial route. Bcl-2 mRNA **(A)** and **(B)** Bax mRNA levels, as determined with qRT-PCR. **(C)** Bax and Bcl-2 proteins. **(D)** Bax/Bcl-2 ratio. **(E)** Bax and cyt *c* in 769-P cells. All results are presented as mean ± SD (*n* = 3). *p < 0.05; **p < 0.01, vs the control. **(F)** Cytosolic proteins were isolated and analyzed by Western blot to measure the localization of Bax and cyt c.

It is known that Δψm is controlled by the Bcl-2 proteins. Therefore, the expressions of Bax and Bcl-2 were assayed using quantitative RT-PCR and Western blot. Results showed that the expression of Bcl-2 was significantly decreased at both mRNA and protein levels (Figures 5A,C). In contrast, Cos treatment significantly increased the mRNA and protein expressions of Bax (Figures 5B,C). Moreover, Bax/Bcl-2 was elevated in Cos-exposed cells, relative to control, indicating enhancement of occurrence of apoptosis (Figure 5D). In addition, Cos enhanced transfer of Bax from the cytosol to the mitochondrion, and enhanced the release of cytochrome *c* from mitochondria (Figures 5E,F). Thus, Cos provoked apoptosis through the mitochondrial pathway.

### Cos Induced Autophagy in RCC Cells

**FIGURE 6:**
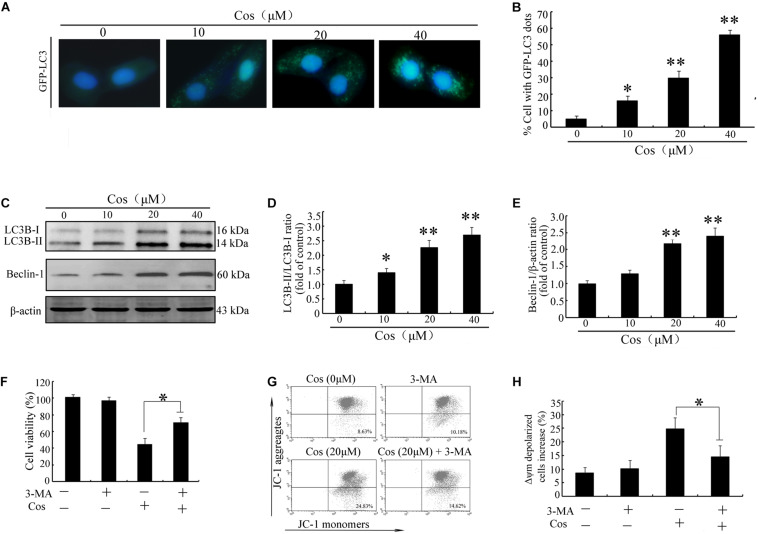
Cos exerted autophagy-mediated cell death through ROS production in 796-P cells. **(A)** Photomicrograph of cells showing GFP-LC3 and indicating formation of autophagosomes. Cos-exposed cells had a punctate profile of GFP-LC3B expression. **(B)** Protein expressions of LC3B-I/lC3-II and Beclin-1, as assayed using Western blot. **(C)** LC3-II/LC3-I ratio and **(D)** Beclin-1/β-actin ratio in Cos-treated 769-P cells, as determined with Image J. **(E)** Cell viability, as measured using CCK8. **(F)** Mitochondrial membrane potential. All results are presented as mean ± SD (*n* = 3). *p < 0.05; **p < 0.01, vs the control. **(G)** The mitochondrial membrane potential were measured using JC-1 staining by flow cytometry. **(H)** The histograms indicate the ratio of green in JC-1 fluorescence.

To determine whether Cos induced autophagy in 769-P cells, GFP-LC3 dot formation was performed. The results showed that Cos treatment accentuated GFP-LC3 puncta generation in 769-P cells in a dose-based fashion (Figures 6A,B). Moreover, the expression of several protein biomarkers of autophagy were assayed with Western blot analysis. The results revealed that Cos treatment increased the protein expressions of LC3B II and Beclin-1 (Figures 6C–E). It is known that autophagy may exert protective effect on cells or contribute to apoptosis (16). Treatment of 769-P cells with 3-MA, an autophagic suppressor, resulted in marked increase in viability (Figure 6F). Furthermore, 3-MA markedly decreased Cos-induced mitochondrial depolarization (Figures 6G,H). These results suggest that inhibiting autophagy could attenuate apoptosis induced by Cos in 769-P cells.

### Cos Induced Autophagy-Associated Cell Death Through ROS

**FIGURE 7:**
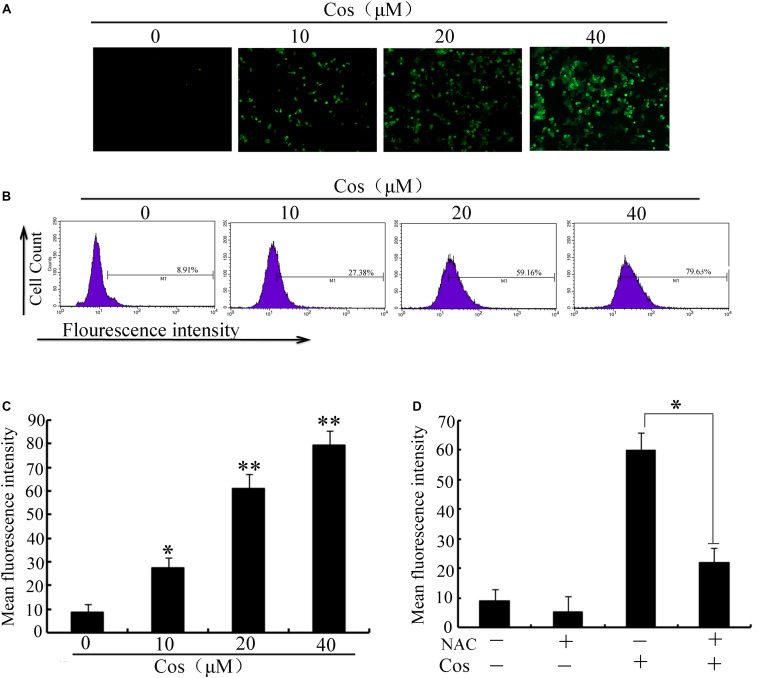
Cos increased ROS generation in 769-P cells. **(A)** ROS levels. **(B)** ROS levels measured as fluorescent intensity. **(C)** ROS of cells pretreated with NAC prior to Cos exposure for 24 h. The histograms indicate the change of fluorescent intensity in Cos treated cells. **(D)** Cells were pretreated with NAC (5 mM) for 2 h and then treated with Cos for 24 h. Fluorescent intensity was detected using flow cytometry. All results are presented as mean ± SD (*n* = 3). *p < 0.05; **p < 0.01, vs the control.

**FIGURE 8:**
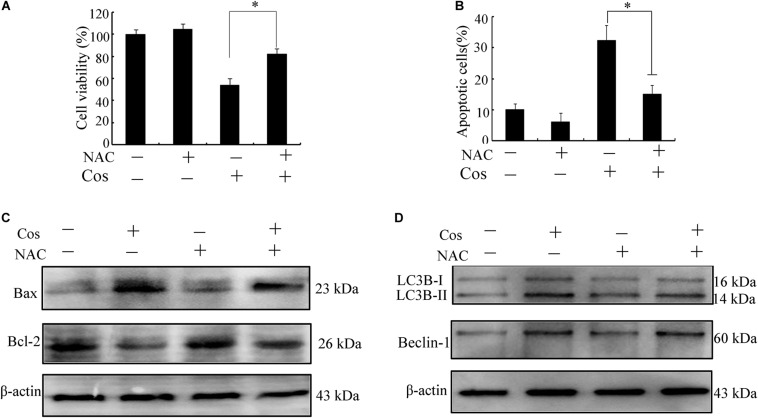
Cos exerted apoptotic and autophagic effects in 769-P cells *via* ROS generation. **(A)** Cell viability. **(B)** Cell apoptosis was evaluated by flow cytometry. **(C,D)** Protein expressions of Bax, Bcl-2, Beclin-1, and LC3B-I/LC3B-II ratio. All results are presented as mean ± SD (*n* = 3). *p < 0.05; **p < 0.01, vs the control.

It is established that ROS are involved in apoptosis and autophagy (20–22). In this study, ROS generation was determined in 769-P cells with the ROS probe DCFH-DA. Treatment with Cos increased ROS in a concentration-dependent fashion (Figures 7A–C). Furthermore, the increases in ROS were significantly attenuated by pretreating the cells with the ROS scavenger N-Acetyl-cysteine (NAC) (Figure 7D). Moreover, NAC treatment attenuated the decrease in cell viability (Figure 8A) and apoptosis (Figure 8B) caused by Cos treatment, and NAC significantly decreased the levels of Bax and LC3-II and increased Bcl-2 level (Figures 8C,D). Thus, ROS are implicated in autophagy and cell death induced by Cos.

### Cos Induced Apoptotic and Autophagic Changes *via* the JNK Signaling Pathway

**FIGURE 9:**
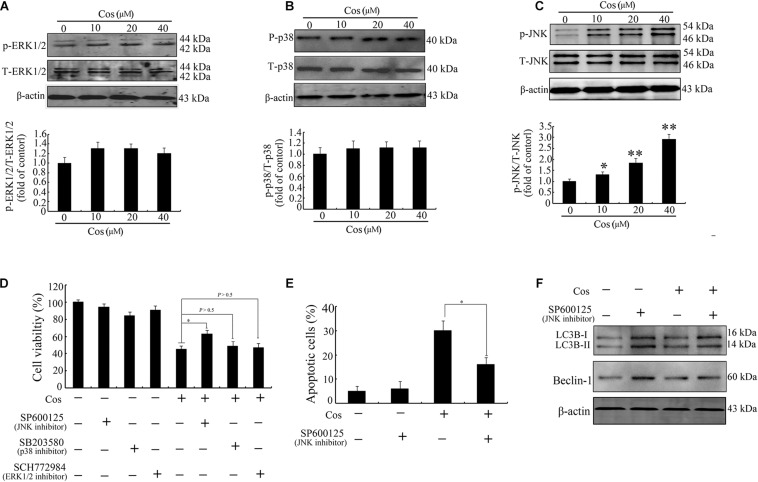
Cos-induced apoptosis and autophagy were associated with JNK activation in 769-P cells. Western-blotting results on protein expressions of ERK1/2 and p-ERK1/2 **(A)**, p38 and p-p38 **(B)**, and JNK and p-JNK **(C)**. Phosphorylated proteins in each immunoblot were normalized to total protein content of the respective protein. **(D)** Results CCK8 assay on 769-P cells pretreated with MEK1/2 inhibitor PD98059 (10 μM), JNK inhibitor SP600125 (10 μM), or the p38 inhibitor SB203580 (10 μM) for 2 h prior to treatment with or without Cos (20 μM), for 24 h. **(E)** Apoptosis of 769-P cells pretreated with JNK inhibitor SP600125 (10 μM) for 2 h prior to treatment with or without Cos (20 μM). **(F)** Expression levels of autophagy-related proteins, as assayed using Western blot. All results are presented as mean ± SD (*n* = 3). *p < 0.05; **p < 0.01, vs the control.

The MAPK signaling route is linked to apoptosis and autophagy (23). Levels of p-ERK1/2, p-JNK, and p38 were assayed in this study. As shown in Figures 9A–C, Cos treatment markedly increased the level of phosphorylated JNK in a concentration-dependent manner. Furthermore, pretreatment with JNK inhibitor (SP600125), but not ERK1/2 inhibitor (SCH772984) or p38 MAPK inhibitor (SB203580), significantly attenuated cell viability caused by Cos treatment (Figure 9D). The Cos-stimulated apoptosis was mitigated by SP600125 (Figure 9E). In addition, SP600125 decreased the protein expressions of LC3-II and Beclin-1 (Figure 9F). Taken together, these results indicate the involvement of JNK in Cos-mediated autophagy and cell death.

### ROS Production Preceded JNK Stimulation in Cos-Provoked Apoptosis

**FIGURE 10:**
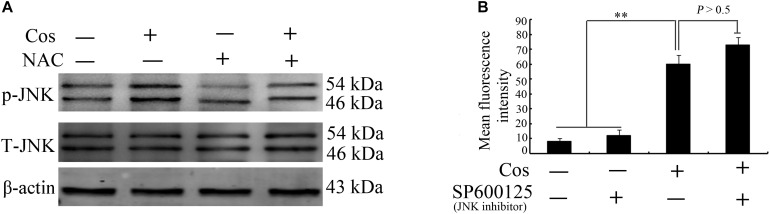
ROS production preceded JNK activation in Cos-provoked apoptosis and autophagy in 769-P cells. **(A)** Effect of NAC (5 mM) on expressions of phospho-JNK1/2 and total JNK1/2 in 769-P cells exposed to Cos (20 μM) for 24 h. **(B)** ROS levels in 769-P cells pretreated with SP600125 (10 μM) for 2 h prior to treatment with or without Cos (20 μM). All results are presented as mean ± SD (*n* = 3). *p < 0.05; **p < 0.01, vs the control.

**FIGURE 11:**
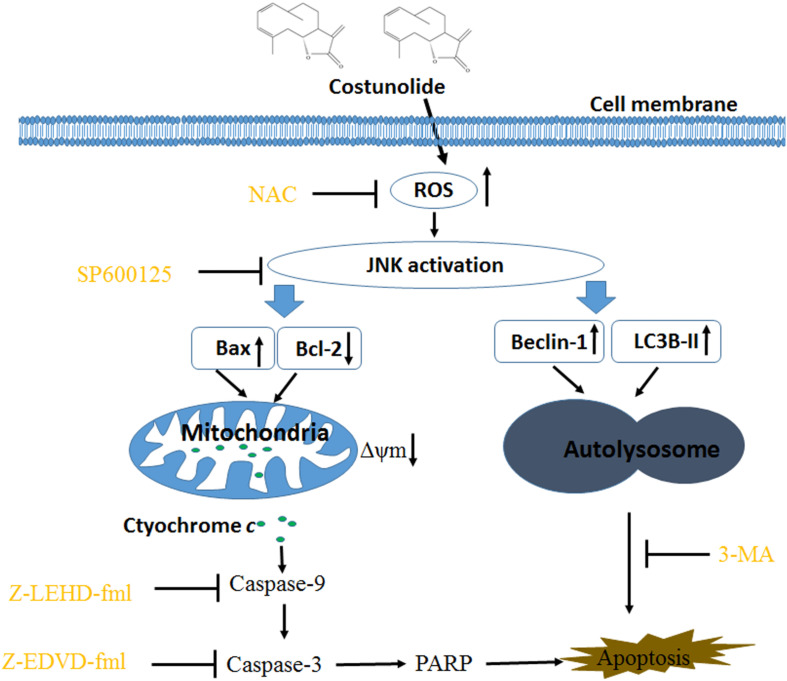
Costunolide induced autophagy and apoptosis via ROS/JNK signaling pathway.

Figure 10A shows that the JNK inhibitor SP600125 did not affect Cos-induced ROS generation, suggesting that JNK did not enhance ROS levels. Interestingly, suppression of ROS with NAC eliminated Cos-associated JNK2 phosphorylation (Figure 10B), indicating that ROS generation preceded the activation of JNK in Cos-treated 769-P cells. Taken together, these findings indicate that ROS/JNK pathway activated by Cos treatment is involved in the induction of apoptosis and autophagy (Figure 11).

## Discussion

Cos is a sesquiterpene lactone isolated from the stem bark of *M. sieboldii*. It exhibits various biological and immunological properties. Previous studies showed that Cos exerted various anticancer effects such as blockage of the angiogenic factor (VEGFR) signaling pathway (24), disruption of microtubule proteins (25), inhibition of telomerase activity (26), and triggering of apoptosis and arrest of the cell cycle (9). However, the association between Cos-induced cell death and autophagy has not been reported. The present study has provided evidence, indicating that Cos induced apoptosis and autophagy in human renal cancer cells *via* ROS/JNK signaling pathway.

It is well-known that apoptosis is a basic event needed for maintenance of tissue constancy (11). Earlier reports have shown that Cos induced apoptotic cell death in different cancers such as breast, lung, bladder, and esophageal cancers (9, 27–30). Consistent with these reports, the results obtained in this study showed that Cos decreased RCC cell viability and increased cell death. Chromatin condensation and presence of phosphatidylserine on the exterior of the cell are crucial indices of apoptosis. These features were present in RCC cells 24 h after Cos treatment, indicating that Cos induced RCC cell apoptosis.

The possible mechanisms underlying Cos-induced apoptosis in 769-P cells were investigated. Cascade activation of caspases plays important roles in apoptosis. The two major caspase activation pathways (death receptor and mitochondrial pathways) have been well described (12). The death receptor pathway is initiated by binding of ligands to death receptors, resulting in caspase-8 activation (13). Mitochondria pathway depends on Cyt c release from mitochondrion into the cytoplasm, leading to caspase-9 stimulation and activation of capase-3 associated with generation of typical apoptotic features. Previous studies showed that Cos induced cancer cell death *via* stimulation of caspase-8 or caspase-9, depending on cancer cell types or other factors. For instance, it has been reported that Cos induced apoptosis of breast and leukemia cancer cells through the extrinsic route (9, 31). Moreover, Cos induced apoptosis in bladder and lung cancer cells through the mitochondrial pathway (28, 29). The results of the present study revealed that Cos promoted caspases-9 and -3, and cleaved PARP in 769-P cells, but caspase-8 was not affected. In addition, the caspase-8 specific inhibitor (z-IETD-fmk) did not attenuate cell death induced by Cos treatment. Western blot and other assays revealed that Cos treatment enhanced Bax/Bcl-2, reduced mitochondrial membrane potential, and resulted in cytochrome *c* release from mitochondrion into the cytosol. These data indicate that Cos induced apoptosis in 769-P cells through mitochondrial pathway.

Protracted exposure of cancerous cells to chemotherapy makes them resist apoptosis. Previous studies have demonstrated that modulation of autophagic processes could be useful for circumventing chemoresistance and enhancing the effects of chemotherapeutics (32, 33). Autophagy is a cellular process for clearance of damaged organelles, and it is involved in carcinogenesis and sensitivity of cancer to therapy (16). Due to different cell types as well as genetic factors, autophagy performs dual roles in cancers. On the one hand, tumor cells can activate autophagy to survive under metabolic and therapeutic conditions by limiting tumor necrosis and mitigating genome damage, such that cancer fighting strategy can be improved by blocking autophagy (15, 34, 35). On the other hand, autophagy may be beneficial in treatment of insensitive cancers (36). In recent years, a great variety of natural products or chemotherapeutic drugs have been demonstrated to participate in the modulation of autophagy through different molecular mechanisms (17, 36–38). For instance, hernandezine, an alkaloid, mediated autophagy in drug-resistant fibroblasts or cancer cells *via* direct stimulation of AMPK (39). Pirarubicin induced an autophagic cytoprotective response *via* inhibition of mTOR/p70S6K signal route in human bladder carcinoma (40). In this study, the results showed that Cos-induced autophagy was evidenced by the increased autophagic vesicle formation and LC3 conversion in 769-P cells. Moreover, 3-MA decreased Cos-provoked cell death, suggesting that autophagy due to Cos was involved in the cell death. These results suggest that Cos induced pro-apoptotic autophagy in 769-P cells.

ROS have been identified as important molecules in the regulation of cell survival or cancer cell death (29, 36). Low ROS concentrations participate in cellular signaling, whereas excessive ROS impair proteins and DNA in the cell, eventually causing autophagy or cell death (32, 41). This study has shown that Cos induced significant increases in ROS, but pretreatment with NAC markedly reversed Cos-associated apoptosis and autophagy, indicating that Cos exerts apoptotic and autophagic influences through the generation of ROS in 769-P cells. It is well-known that ROS, acting as second messengers, exert their biological effects *via* activation of downstream molecules, mainly MAPK signaling pathways (21, 42, 43). Cinobufagin exerted apoptotic and autophagic cell death *via* the ROS/JNK/p38 signaling pathway (44). The ROS-mediated JNK signal route can also modulate autophagic cyto-protection in Ciclopirox olamine-administered rhabdomyosarcoma (45). The results of this study are consistent with these reports, in that among the members of the MAP kinase family studied, only JNK, but not ERK or p38 was activated in Cos-treated 769-P cells. The JNK inhibitor SP600125 significantly reversed Cos-mediated apoptotic and autophagic lesions. In addition, the stimulation of JNK pathway regulated Beclin-1 expression thereby triggering autophagy. Consistent with this finding, Cos significantly increased Beclin-1 expression, and the increases in Becline-1 expression were blocked by SP600125. These results showed that prior NAC exposure attenuated p-JNK. These findings indicate that Cos induced apoptotic and autophagic changes *via* the activation of ROS/JNK signal route.

In summary, the results of this study have demonstrated that Cos treatment exerted apoptotic and autophagic effects *via* ROS/JNK-linked signaling route. Therefore, Cos could be a novel antitumor drug candidate for therapy of renal cell carcinoma and other cancers.

## Data Availability Statement

All datasets presented in this study are included in the article/supplementary material.

## Author Contributions

DF, ZYX, and DW developed the project. DF, ZYX, WC, and JGa performed experiments and wrote the manuscript. JGe, ZYZ, and WQZ supervised the work. All authors read and approved the final manuscript.

## Conflict of Interest

The authors declare that the research was conducted in the absence of any commercial or financial relationships that could be construed as a potential conflict of interest.
